# Primary Cauda Equina Lymphoma Mimicking Meningioma

**DOI:** 10.3390/jcm13164959

**Published:** 2024-08-22

**Authors:** Pierfrancesco Lapolla, Vincenza Maiola, Pietro Familiari, Gabriella Tomei, Dominella Gangemi, Sara Ienzi, Roberto Arcese, Mauro Palmieri, Michela Relucenti, Andrea Mingoli, Gioia Brachini, Stefania Annarita Nottola, Giancarlo D’Andrea, Biagia La Pira, Placido Bruzzaniti

**Affiliations:** 1 Nuffield Department of Surgical Sciences, John Radcliffe Hospital, Headington, University of Oxford, Oxford OX1 2JD, UK; 2 Division of Neurosurgery, Fabrizio Spaziani Hospital, 03100 Frosinone, Italy; vincenza.maiola@aslfrosinone.it (V.M.); giancarlo.dandrea@aslfrosinone.it (G.D.); biagia.lapira@aslfrosinone.it (B.L.P.); placido.bruzzaniti@uniroma1.it (P.B.); 3 Department of Human Neurosciences, Division of Neurosurgery, Policlinico Umberto I University Hospital, Sapienza University of Rome, 00157 Rome, Italy; pietro.familiari@uniroma1.it (P.F.); mauro.palmieri@uniroma1.it (M.P.); 4 Division of Haematology, Bone Marrow Transplantation and Gene Therapy, Fabrizio Spaziani Hospital, 03100 Frosinone, Italy; gabriella.tomei@aslfrosinone.it (G.T.); dominella.gangemi@aslfrosinone.it (D.G.); 5 Department of Pathology, Fabrizio Spaziani Hospital, 03100 Frosinone, Italy; sara.ienzi@aslfrosinone.it (S.I.); roberto.arcese@aslfrosinone.it (R.A.); 6 Department of Anatomy, Histology, Forensic Medicine and Orthopaedics, Sapienza University of Rome, 00185 Rome, Italy; michela.relucenti@uniroma1.it (M.R.); stefania.nottola@uniroma1.it (S.A.N.); 7 Department of Surgery “Pietro Valdoni”, Sapienza University of Rome, 00185 Rome, Italy; andrea.mingoli@uniroma1.it (A.M.); gioia.brachini@uniroma1.it (G.B.)

**Keywords:** primary cauda equina lymphoma, neurolymphomatosis, diffuse large B-cell lymphoma, spinal cord lesions, diagnostic challenges, treatment outcomes

## Abstract

**Background**: Spinal cord lymphomas represent a minority of extranodal lymphomas and often pose diagnostic challenges by imitating primary spinal tumors or inflammatory/infective lesions. This paper presents a unique case of primary cauda equina lymphoma (PCEL) and conducts a comprehensive review to delineate the clinical and radiological characteristics of this rare entity. **Case Report**: A 74-year-old male presented with progressive paresthesia, motor weakness, and symptoms indicative of cauda equina syndrome. Neurological examination revealed paraparesis and sphincter dysfunction. Imaging studies initially suggested an intradural meningioma. However, surgical intervention revealed a diffuse large B-cell lymphoma infiltrating the cauda equina. **Findings**: A systematic review of the pertinent literature identified 18 primary cauda equina lymphoma cases. These cases exhibited diverse clinical presentations, treatments, and outcomes. The mean age at diagnosis was 61.25 years for women and 50 years for men, with an average follow-up of 16.2 months. Notably, 35% of patients were alive at 18 months, highlighting the challenging prognosis associated with PCEL. **Discussion**: Primary spinal cord lymphomas, especially within the cauda equina, remain rare and diagnostically complex due to their nonspecific clinical manifestations. The review highlights the need to consider spinal cord lymphoma in patients with neurological symptoms, even without a history of systemic lymphoma. **Diagnostic Approaches**: Magnetic resonance imaging (MRI) serves as the primary diagnostic tool but lacks specificity. Histopathological examination remains the gold standard for definitive diagnosis. The review underscores the importance of timely biopsy in suspected cases to facilitate accurate diagnosis and appropriate management. **Management and Prognosis**: Current management involves biopsy and chemotherapy; however, optimal treatment strategies remain ambiguous due to the rarity of PCEL. Despite aggressive therapeutic interventions, prognosis remains poor, emphasizing the urgency for enhanced diagnostic and treatment modalities. **Conclusions**: Primary cauda equina lymphoma poses diagnostic and therapeutic challenges, necessitating a high index of suspicion in patients with atypical spinal cord symptoms. Collaborative efforts between neurosurgical, oncological, and infectious diseases teams are imperative for timely diagnosis and management. Advancements in diagnostic precision and therapeutic options are crucial for improving patient outcomes.

## 1. Introduction

Spinal cord lymphomas contribute to approximately 1–2% of extranodal lymphomas and often manifest as intrathecal epidural lesions [1,2]. The identification of these tumors poses a significant challenge due to their propensity to mimic primary spinal tumors and inflammatory or infective lesions. This diagnostic complexity often leads to an initial dismissal of potential suspects, resulting in inconsistent diagnostic findings. In this paper, we present our case of primary cauda equina lymphoma, accompanied by a comprehensive review of the existing literature. Our objective is to examine the clinical and radiological characteristics associated with primary cauda equina lymphoma, particularly those that manifest as masses or mass-like lesions. Primary cauda equina lymphoma (PCEL) is a distinct subtype of neurolymphomatosis that manifests as a primary lesion within the cauda equina. This subtype of lymphoma is extremely rare, with only a few cases reported in the literature. Neurolymphomatosis is an unusual lymphoma manifestation, characterized by the infiltration and destruction of peripheral nerves, spinal nerve roots, nerve plexuses, and cranial nerves by lymphoma cells with neurotropism [3]. Histologically, B-cell lymphomas constitute the majority of neurolymphomatosis cases, with frequencies of B-cell involvement documented as high as 82% and 97.5% in previous studies. T-cell lymphomas, although less common, have been reported in 2.5% to 10% of cases [4]. Neurolymphomatosis usually presents due to secondary involvement from systemic lymphoma, making primary neurolymphomatosis extremely rare. It is estimated that PCEL accounts for less than 1% of neurolymphomatosis cases. This paper describes a unique case in which a diffuse large B-cell lymphoma affected multiple nerve roots in the cauda equina. The tumor occupied the lower spinal canal and presented as a large tumor.

## 2. Report

The patient, a 77-year-old male, presented with a four-month history of progressive paresthesia in his legs and perianal region, with significant motor weakness in both lower limbs primarily affecting the proximal muscles. The patient presented with symptoms of cauda equina syndrome (CES) characterized by dysfunction of multiple lumbar and sacral nerve roots of the cauda equina leading to sphincter dysfunction, urinary urgency, and fecal retention. The patients’ medical history revealed the presence of arterial hypertension, obliterating arteriopathy of the lower limbs, bilateral coronary artery bypass surgery, and carotid endarterectomy.

### 2.1. Clinical Neurological Presentation

The neurological examination revealed paraparesis with a Medical Research Council (MRC) Scale 4/5 in the left lower limb and MRC 3/5 in the right lower limb, particularly in the proximal muscles, mild hypoesthesia or paresthesia in both legs and the perianal region, and anal sphincter hypertonus. Areflexia was observed in the lower limbs, although muscle stretch reflexes were normal in other areas.

### 2.2. Imaging Findings and Additional Diagnostics

Magnetic resonance imaging (MRI) of the thoracic–lumbar–sacral spine was performed using conventional biplanar sequences, both with and without fat saturation, before and after the administration of a gadolinium-based contrast agent (ProHance^®^ Bracco Diagnostics Inc., Milan, Italy). The imaging focused on the vertebral canal, particularly at the D12 and L1 levels.

**Figure 1:**
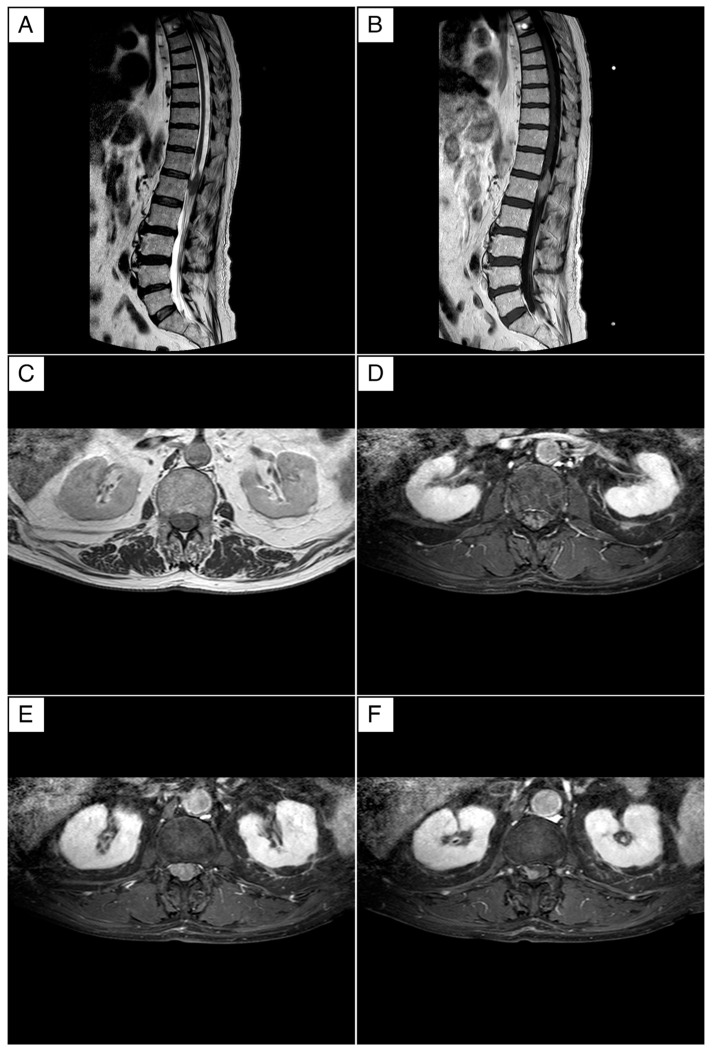
Magnetic resonance imaging (MRI) of the dorso–lumbar spine. (**A**) The T2-weighted sagittal MRI demonstrates an extra-axial lesion exhibiting a hypointense signal. (**B**) The T1-weighted sagittal MRI demonstrates a lesion exhibiting a hyperintense signal. (**C**) The T2-weighted axial MRI demonstrates a structure that compresses but appears to not infiltrate the cauda. (**D**–**F**) Gadolinium-enhanced T1-weighted axial MRI shows an extra-axial lesion with homogeneous signal enhancement. This lesion appears to be of extra axial origin, and the characteristics of signal and enhancement can be attributed in the initial hypothesis to a meningioma with a probable short right anterolateral implant in accordance with an image attributed to a dural tail.

The MRI identified a large intraspinal, extra-axial lesion at the D12/L1 level, measuring approximately 36 mm in longitudinal diameter (Figure 1A,B). On T1-weighted sequences, the lesion appeared hyperintense, while on T2-weighted sequences, it was hypointense. Post-contrast images showed homogeneous signal enhancement, confirming its solid nature and measuring about 36 mm. These imaging characteristics suggested the lesion was an extra-axial meningioma, likely with a short right anterolateral dural tail implant, indicative of a dural tail sign.

The lesion was compressing the spinal cord without infiltrating the cauda or the dura mater, as shown in Figure 1C–F. Despite the compression, there was no evidence of infiltration into the surrounding structures. The radiology department’s head, with over 25 years of experience, reviewed the MRI findings and confirmed the initial diagnosis of an intraspinal intradural meningioma.

### 2.3. Operative Findings and Histopathology

The patient underwent microsurgical tumor resection via a posterior thoracolumbar approach (D12-L2 laminectomy) and a midline durotomy. The tumors’ appearance was unexpectedly different: a soft, crumbly mass with diffuse adherence to the dura and infiltrations of intradural structures (conus medullaris and nerve roots). Due to the heightened risk of neurological injuries upon discovering the tumor’s diffuse adherence to the dura and intradural structures, a biopsy of the lesion was performed instead of a complete resection. The intraoperative somatosensory-evoked potentials remained equal to the preoperative baseline throughout the entire surgical procedure.

**Figure 2:**
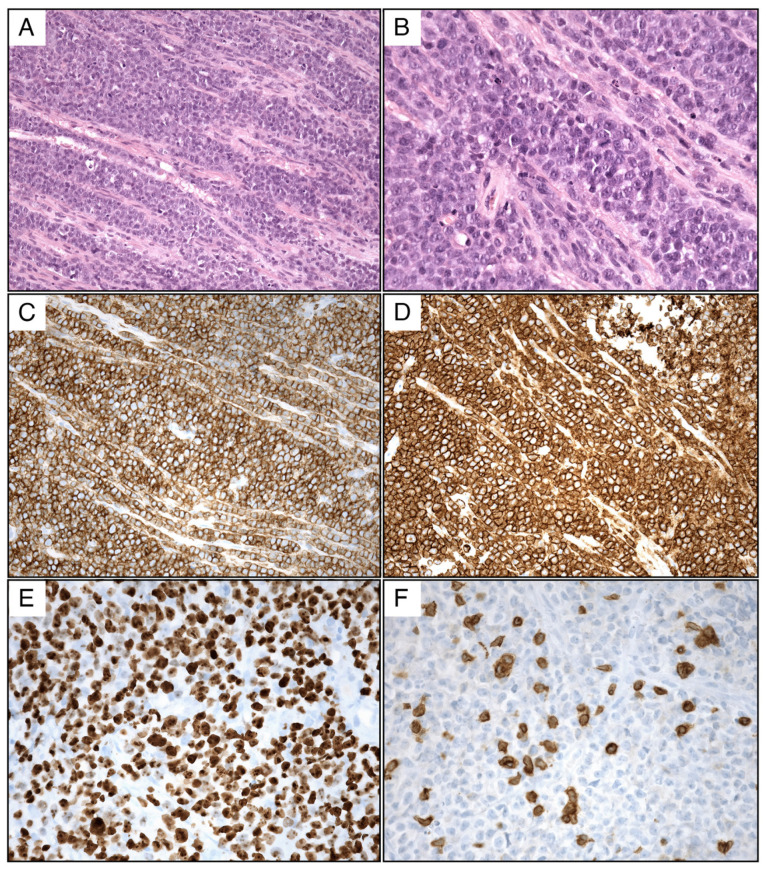
Histologic examination of primary cauda equina diffuse large B-cell lymphoma (DLBCL) (**A**) H&E 20X Bundles of nerve fiber infiltrated by DLBCL. The neoplasm has a diffuse and starry-sky pattern; (**B**) H&E 40X lymphoma cells are large and round. Mitotic figures and apoptotic bodies are present; (**C**) LCA 20X lymphoma cell are strongly positive for LCA; (**D**) CD20 20X lymphoma cells are positive for CD20, supporting B-cell lineage; (**E**) Ki67 40X lymphoma cells have a high proliferation rate (Ki67); (**F**) CD3 40X lymphoma cells are negative for CD3.

The microscopic examination of the tissue samples, as shown in Figure 2, revealed a pattern of diffuse non-germinative extranodal large-cell lymphoma as per the HANS algorithm (BLOOD 2004), characterized by a high proliferation index of 90% Ki67. The immunophenotype of the peripherally derived B-cell lymphoma was characterized by positive CD20 and CD79a and negative CD3 and CD5, Cycline D1-, CD10-, Bcl6+, Bcl2+, MUM1+, PAX5+, TdT-, and c-myc +> 40%. In situ hybridization (FISH) was performed, and it was found to be negative for the translocation t(14; 18), for the BCL6 mutation, and the rearrangement of the Myc gene on the chromosomal region 8q24. Unfortunately, cerebrospinal fluid was not examined.

### 2.4. Postoperative Recovery

Postoperatively, the patient’s neurological status remained unchanged. The sensory and motor deficits persisted, and the sphincter function remained unchanged. A comprehensive whole-body CT scan conducted subsequent to the operation revealed that the cauda lesion remained unaffected by any postoperative complications. The patient was transferred to the oncology department and underwent seven cycles of an aggressive chemotherapy regimen that included Bendamustine (an alkylating agent) and Rituximab (a monoclonal antibody), commencing 18 days after surgery. Each cycle was administered at intervals adjusted to the patient’s recovery and tolerance levels. This treatment approach, based on the current understanding of managing primary CNS lymphomas, led to complete remission with no residual symptoms. The patient was moved to the infectious diseases department due to the development of severe bacterial meningitis following chemotherapy, which required specialized care to manage this serious infectious complication. This intervention was critical to stabilizing the patient and preventing further deterioration of his condition.

### 2.5. Management in the Infectious Diseases Department and Follow-Up

Upon transfer to the infectious diseases department, the patient was carefully evaluated for any potential infections that could exacerbate his condition. Efforts to prevent infection were taken, taking into account his immunocompromised state resulting from chemotherapy. The patient was also closely monitored for signs of neurotoxicity, which is a common side effect of both the lymphoma and the chemotherapy regimens. Regular neurological examinations were conducted to assess any changes in his sensory and motor functions.

### 2.6. Subsequent Oncological Treatment

After a brief period of stabilization in the infectious diseases department, the patient was moved back to the oncology department. The patient completed a total of seven cycles of Bendamustine and Rituximab chemotherapy. During the treatment period, imaging studies showed significant reduction in the cauda equina lesion size. Symptomatically, the patient experienced progressive improvement in sensory and motor functions. The total observation period extended over 12 months, and at the time of the last follow-up, the patient was in complete remission with no evidence of disease recurrence and stable neurological function.

The response to treatment was evaluated using regular imaging studies. Periodic full-body CT scans were performed to assess the regression of the cauda lesion and other tumor localizations. The patient’s condition, including potential development of new symptoms, side effects of the treatment, and overall physical health, was continually monitored.

### 2.7. Long-Term Follow-Up and Prognosis

After completing the chemotherapy cycles, the patient was scheduled for regular follow-up visits to monitor his recovery and check for any signs of recurrence. These visits included a comprehensive clinical examination, blood tests to monitor his health status, and imaging studies to detect any potential relapse early.

Despite the aggressive nature of the diffuse large B-cell lymphoma and the complexity of the patient’s case, the interdisciplinary approach to his treatment and careful management of his symptoms allowed for an optimistic prognosis. While his long-term prognosis remained uncertain due to the rarity and aggressive nature of PCEL, the combined efforts of the neurosurgical, oncological, and infectious diseases teams provided the patient with the best possible care and improved his quality of life.

## 3. Materials and Methods

We conducted an extensive and thorough systematic review of the pertinent literature. This review was restricted to articles that were peer-reviewed, ensuring a high level of credibility and reliability in the research. The review period was extended up until September 2023.

For the purpose of this review, we utilized two major electronic databases, namely SCOPUS and PUBMED. To refine our search and focus on the most relevant articles, we employed Boolean operators, “AND” and “OR”. The search terms we used included “Primary cauda equina diffuse large B cell Non-Hodgkin lymphoma”. These terms were used in various combinations to capture the broadest possible range of relevant articles.

Our initial search for the keyword combination resulted in a total of 229 articles. After recognizing the need to narrow down the search to articles specifically addressing lymphomas of the cauda equina, a refined search yielded a more manageable total of 51 articles.

Each of these articles was thoroughly screened by their title, abstract, and full text. This allowed us to identify the most relevant case studies and literature reviews, which were subsequently sourced and studied in detail. This process produced a final selection of 18 articles.

Any disagreements or conflicts that arose during the article selection process were resolved through discussion and consensus. To ensure the relevance and accuracy of our review, we excluded any articles not written in English and those representing duplicate cases reported in multiple articles.

## 4. Results

**Figure 3:**
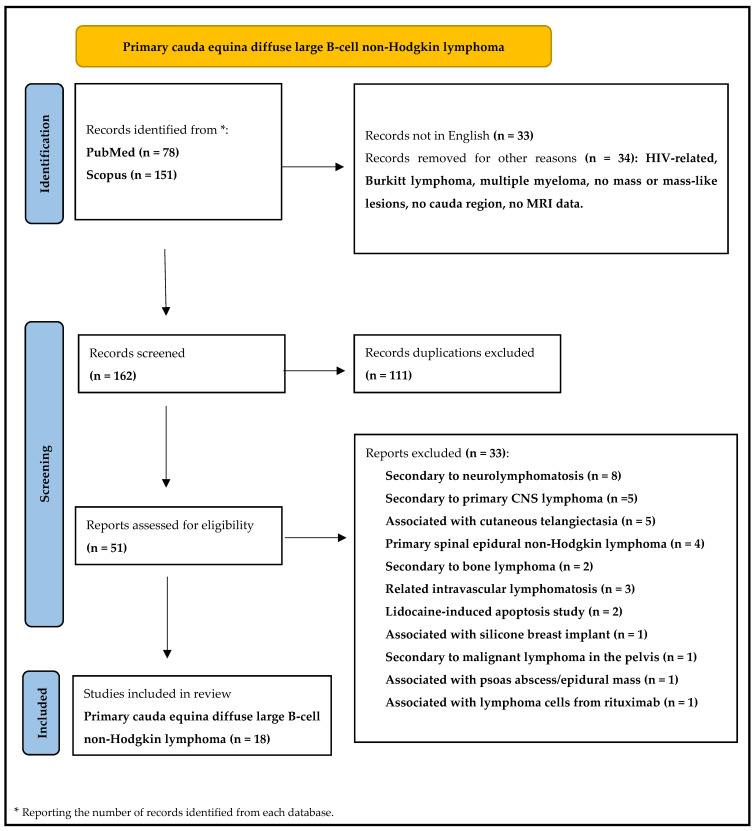
Flow diagram for new systematic reviews of primary cauda equina diffuse large B-cell non-Hodgkin lymphoma.

A detailed algorithm of our literature search is provided in Figure 3 for further understanding of our search process. We meticulously reviewed the selected articles and extracted essential patient characteristics, including age, gender, clinical presentation, tumor location, management strategy, and clinical outcome. These cases were systematically categorized into primary and metastatic spinal cord lymphomas, with the detailed analysis and interpretation of these data presented in Table 1 and Table 2. This organization ensures a clear distinction between the procedural aspects and findings of our study.

From our systematic review, we identified 18 cases of primary cauda equina lymphoma. The mean age at diagnosis was 61.25 years for women and 50 years for men. The follow-up duration varied, with an average of 16.2 months. Survival data indicated that 35% of patients were alive at 18 months post diagnosis.

In terms of clinical management, 11% of the cases involved a cytoreductive operation aimed at reducing tumor burden, while the remaining cases predominantly involved biopsies to confirm the diagnosis. Treatment modalities varied significantly: 27% of patients received intravenous therapy, while 44% underwent cerebrospinal fluid (CSF) examination or radiotherapy. These differences reflect the individualized treatment approaches based on tumor characteristics, patient health, and clinician preference.

Patients commonly presented with symptoms indicative of cauda equina syndrome (CES), which included lower-back pain, bilateral lower extremity weakness, sensory deficits, and sphincter dysfunction. This clinical picture often led to initial misdiagnoses, complicating and delaying appropriate treatment.

MRI findings were consistent across cases, typically showing lesions at the level of the cauda equina that were hyperintense on T1-weighted sequences and hypointense on T2-weighted sequences. These lesions often exhibited heterogeneous contrast enhancement. The radiological features, while suggestive of a mass, were insufficient alone to differentiate lymphoma from other potential diagnoses such as meningiomas or metastatic disease, underscoring the necessity of histopathological confirmation.

The treatment regimens varied, reflecting the complexity and rarity of the condition. Biopsy procedures were the most common initial intervention to obtain a definitive diagnosis. Following histopathological confirmation, treatment approaches included the following:

- Intravenous chemotherapy: administered to 27% of patients, aimed at systemic disease control;
- Radiotherapy: applied in 44% of cases, targeting the tumor localized within the spinal canal;
- Surgical intervention: cytoreductive surgeries performed in 11% of cases, aimed at decompressing neural structures and alleviating symptoms.

Survival outcomes were variable, with a mean follow-up period of 16.2 months. At the 18-month mark, 35% of patients were alive, indicating the potential for long-term survival with appropriate treatment. However, the overall prognosis remained guarded due to the aggressive nature of lymphoma and the critical location affecting neural functions.

## 5. Discussion

Over the past four decades, there has been a noticeable increase in the incidence of non-Hodgkin lymphoma (NHL) in the United States [22,23]. This rise can be attributed to an enhanced understanding of NHL’s pathogenesis, advancements in diagnostic modalities, and the development of more efficacious anti-neoplastic agents. NHL of the central nervous system (CNS) can affect various areas, including the brain parenchyma, leptomeninges, spinal cord, spinal nerve roots, or epidural space. While primary or secondary NHL in the brain and meninges is more common, occurrences in the intramedullary spinal cord remain rare. The spinal cord is more frequently affected by an epidural primary or secondary deposit of NHL within the spinal canal [24].

Conversely, systemic Hodgkin lymphoma infrequently affects the CNS, with a prevalence between 0.2% and 0.5% [25]. To the best of our knowledge, there are no documented cases in the literature reporting Hodgkin lymphoma affecting the intramedullary spinal cord. Similarly, T-cell lymphoma rarely affects the spinal cord, given the shortage of cases reported in the literature [26,27]. Therefore, our discussion primarily focuses on NHL affecting the spinal cord. Our comprehensive review of the literature suggests that NHL can impact all levels of the spinal cord, including the cauda equina.

### 5.1. Clinical Presentation

Spinal cord lymphomas often present with symptoms such as weakness, sensory loss, spasticity, pain, and bowel or bladder incontinence. In our case, the patient presented with non-specific neurological symptoms. Several factors contributed to the low suspicion of lymphoma. First, the patient did not exhibit a prior history of weight loss, loss of appetite, or the typical “B” symptoms often associated with lymphoma. Second, the patient was not immunocompromised, a common characteristic in lymphomas affecting extranodal sites. Third, the clinical presentation resembled that of an inflammatory disease or primary spinal cord tumor, such as an ependymoma or astrocytoma.

Our literature review reveals no discernible disparity in the nature of symptoms or severity between primary and secondary spinal cord lymphomas. As such, we propose that spinal cord lymphoma should be considered a differential diagnosis in all patients presenting with neurological symptoms related to the spinal cord, even in cases where the patient does not have a history of systemic lymphoma.

Primary cauda equina lymphoma presents a diagnostic challenge due to its rarity and nonspecific symptoms. The clinical presentation often mimics other, more common spinal pathologies such as meningiomas, metastases, or inflammatory conditions. Our case of a 77-year-old man with progressive lower-back pain, bilateral lower extremity weakness, and bowel and bladder dysfunction is consistent with the presentation of cauda equina syndrome (CES). Similar cases reported in the literature describe patients presenting with CES characterized by sphincter dysfunction, urinary urgency, and fecal retention. The average age of presentation varied, with a mean age of 61.25 years for women and 50 years for men, reflecting a slight predilection for older adults.

### 5.2. Diagnostic Approaches

MRI of the brain and spine is the preferred imaging modality and the first investigation of choice when a CNS lymphoma is suspected. The most common form of presentation of intramedullary spinal cord lymphomas on MRI is a solitary lesion. However, multifocal presentation is more commonly found in immunocompromised patients. The reported signal characteristics included an hyperintense signal to the spinal cord in T1 and hypointense in T2 and STIR signal (Short T1 Inversion Recovery), which is a characteristic deviation from the low T2 intensity seen in intracranial lesions. In most cases, lesions homogenously enhance, but sometimes, the enhancement may be heterogeneous. The distribution of these lesions within the spinal cord is predominantly central, similar to the central distribution seen in astrocytoma and ependymomas [11]. Despite its benefits, MRI cannot provide a definitive diagnosis of spinal cord lymphoma, as its imaging characteristics overlap with other pathologies, such as demyelinating diseases, vascular malformations, and other neoplasms. Therefore, histopathological examination remains the gold standard for definitive diagnosis.

### 5.3. Differential Diagnosis

The diagnosis of primary cauda equina lymphoma is often delayed due to its rarity and the overlap of its imaging features with other spinal tumors. MRI findings in our case showed a hyperintense lesion on T1-weighted sequences and a hypointense lesion on T2-weighted sequences with heterogeneous contrast enhancement. These imaging characteristics are not unique to lymphoma and can be seen in other pathologies such as meningiomas or metastases. Histopathological examination remains the gold standard for diagnosis. Our review of the literature corroborates this, as many cases required biopsy for definitive diagnosis. The involvement of experienced radiologists and pathologists is crucial in the timely and accurate diagnosis of this condition. The differential diagnosis for spinal cord lymphoma includes other primary intramedullary neoplasms such as astrocytoma and ependymomas, metastatic deposits, vascular malformations like cavernomas, inflammatory diseases like sarcoidosis, and demyelinating diseases such as transverse myelitis or multiple sclerosis. Each of these conditions has distinct imaging characteristics on MRI, which can aid in narrowing down the differential diagnosis. However, due to the high degree of overlap in imaging characteristics between these conditions and lymphoma, a definitive diagnosis can only be confirmed with histopathological examination. Therefore, any patient with an MRI suggestive of spinal cord lymphoma should undergo a biopsy for definitive diagnosis.

### 5.4. Histopathological Examination and Diagnosis

Histopathological examination of spinal cord lymphoma typically reveals a dense infiltrate of lymphocytes with large, round, and hyperchromatic nuclei and scant cytoplasm. Immunohistochemistry often shows that these cells are B lymphocytes that express positive CD20 and CD79a and negative CD3/CD5 and other B-cell markers.

In our case, the tumor was composed of large B-cell lymphoma, which is the most common subtype of NHL. This subtype has a predilection for extranodal sites and often presents with a rapidly growing mass, as was seen in our patient.

### 5.5. Management and Prognosis

Treatment of primary cauda equina lymphoma typically involves a multimodal approach, including surgery, chemotherapy, and radiotherapy. In our case, the patient underwent a biopsy followed by appropriate oncological treatment. Our systematic review found that 11% of cases involved a cytoreductive operation, while biopsies were performed in the remaining cases. Intravenous chemotherapy was administered to 27% of patients, and 44% underwent CSF examination or radiotherapy. The varied treatment approaches reflect the individualized nature of care based on tumor characteristics, patient health, and clinician preference.

Survival outcomes varied significantly among the reviewed cases. The average follow-up duration was 16.2 months, with 35% of patients surviving at 18 months post diagnosis. This highlights the potential for long-term survival with appropriate and timely treatment. However, the overall prognosis remains guarded due to the aggressive nature of lymphoma and its critical location affecting neural functions.

The standard treatment for primary CNS lymphoma is high-dose methotrexate-based chemotherapy, often followed by whole-brain radiotherapy. However, the optimal treatment for primary spinal cord lymphoma remains unclear due to the rarity of this condition.

In our case, the patient underwent a biopsy of the lesion, followed by chemotherapy. This approach was chosen based on the intraoperative findings, which precluded a complete resection due to the risk of neurological damage. The treatment regimen included Rituximab due to the patient’s CD20-positive status, which made this therapy particularly effective. The regimen also incorporated Bendamustine, an alkylating agent. High-dose intravenous chemotherapy was chosen based on its demonstrated efficacy in CNS lymphomas. Radiation therapy as a consolidation stage was considered but not implemented to avoid potential neurotoxicity, considering the patient’s overall health and response to chemotherapy. This approach aligns with findings that systemic chemotherapy, particularly with immunomodulators, is indispensable for treating spinal cord lymphomas, whereas radiation is reserved for refractory cases.

### 5.6. Comparative Analysis

When comparing our case to others in the literature, several similarities and differences emerge. Most cases presented with symptoms of CES, though the exact clinical features varied. Diagnostic imaging consistently revealed intraspinal lesions with contrast enhancement, but the differential diagnosis often included meningiomas and other neoplasms. Histopathological confirmation was universally required for a definitive diagnosis.

The treatment strategies varied widely, with some patients undergoing extensive surgical resection, while others received limited biopsy followed by chemotherapy and/or radiotherapy. These differences underscore the lack of standardized treatment protocols for primary cauda equina lymphoma and highlight the need for further research to establish evidence-based guidelines.

### 5.7. Limitations

This systematic review is limited by several factors that warrant careful consideration. First and foremost, the small number of reported cases of primary cauda equina lymphoma significantly constrains our ability to draw broad conclusions. The rarity of this condition means that available data are sparse, and many published reports are based on single-case studies or small case series. This limited sample size reduces the statistical power of our analysis and hampers the generalizability of our findings.

Secondly, there is considerable heterogeneity in patient management across the reported cases. Treatment strategies varied widely, reflecting differences in clinical practice, patient characteristics, tumor biology, and availability of medical resources. For instance, some patients underwent extensive surgical resection, while others received limited biopsy followed by chemotherapy and/or radiotherapy. This variability complicates the direct comparison of outcomes and the establishment of standardized treatment protocols.

The variability in follow-up durations also presents a significant limitation. Follow-up periods ranged from a few months to several years, affecting the ability to assess long-term outcomes consistently. Shorter follow-up periods may not capture late recurrences or long-term complications, while longer follow-up is necessary to understand the true prognosis of primary cauda equina lymphoma. Additionally, variations in follow-up practices across different institutions and studies contribute to this inconsistency.

Another limitation stems from the retrospective nature of the majority of the included case reports and case series. Retrospective studies are inherently subject to biases, including selection bias, recall bias, and incomplete data collection. These biases can influence the reported outcomes and may not accurately reflect the true clinical picture.

Furthermore, there is a lack of standardized diagnostic criteria and treatment protocols for primary cauda equina lymphoma. The diagnosis often relies on a combination of clinical, radiological, and histopathological findings, but the criteria used can vary between studies. Similarly, treatment approaches are not uniform, with some clinicians favoring aggressive surgical intervention and others opting for conservative management with chemotherapy and radiotherapy. This lack of standardization complicates the interpretation of results and highlights the need for consensus guidelines.

The rarity of primary cauda equina lymphoma also limits the availability of high-quality prospective studies. Most of the evidence is derived from case reports and small case series, which provide valuable insights but lack the methodological rigor of larger, controlled studies. Prospective multicenter studies and randomized controlled trials are needed to provide higher-quality evidence and to inform clinical practice more robustly.

Finally, the literature on primary cauda equina lymphoma is subject to publication bias. Cases with positive outcomes or novel findings are more likely to be reported and published, while negative or less remarkable cases may go unreported. This can skew the overall picture of the disease and its management, leading to an overestimation of treatment efficacy and underestimation of complications.

## 6. Conclusions

In conclusion, primary spinal cord lymphoma, although an exceedingly rare entity, presents a diagnostic challenge due to its nonspecific clinical and radiological manifestations. It is often mistaken for more common spinal cord pathologies, including inflammatory diseases and other types of primary spinal cord tumors. This diagnostic dilemma can result in delayed or inaccurate treatment, impacting patient outcomes significantly. Given the aggressive nature of this disease, early recognition is of paramount importance. This involves maintaining a high index of suspicion in patients presenting with rapidly progressive neurological symptoms, especially when the individual does not fit the usual demographic or clinical profile for a spinal cord tumor.

To this end, a comprehensive diagnostic approach is necessary for accurate and timely diagnosis. MRI is crucial for identifying and characterizing spinal cord lymphomas. Specific radiological features, such as hypointense signals on T1-weighted images and enhancement patterns post contrast, can overlap with other pathologies like meningiomas. However, findings such as diffuse thickening and enhancement of cauda equina nerve roots strongly suggest lymphoma. Given that primary cauda equina lymphoma is a subtype of primary CNS lymphoma, high-dose methotrexate-based strategies were utilized. The role of autologous hematopoietic stem cell transplantation as a consolidation stage, although beneficial in primary CNS lymphomas, remains under investigation for cauda equina lymphoma and was not employed in this case due to specific patient factors.

Hence, histopathological examination, along with immunohistochemical analysis, remains the cornerstone for confirming the diagnosis of spinal cord lymphoma.

Treatment strategies for primary spinal cord lymphoma are still evolving due to its rarity. Currently, biopsy and chemotherapy appear to be the most effective approach. The role of radiotherapy, while beneficial in primary CNS lymphoma, is yet to be clearly defined in the context of spinal cord lymphoma. It is also crucial to consider the patient’s age, overall health status, and potential treatment-related complications when formulating a treatment plan.

Primary cauda equina lymphoma, although rare, should be considered in the differential diagnosis of cauda equina syndrome. Early and accurate diagnosis followed by appropriate management is crucial for optimal patient outcomes. Our case and literature review underscore the necessity for heightened awareness and prompt intervention in such cases. Further research is needed to establish standardized diagnostic and treatment protocols to improve patient prognosis.

Lastly, the prognosis of primary spinal cord lymphoma is generally poor, underscoring the need for further research and advancements in therapeutic options. Multidisciplinary collaboration and ongoing clinical trials are essential in improving our understanding of this rare entity, refining diagnostic strategies, and developing more effective treatment regimens.

In the future, the discovery of specific genetic or molecular markers may offer more targeted therapy options and potentially improve outcomes for patients with this aggressive disease. Until then, clinicians must remain vigilant and consider primary spinal cord lymphoma in the differential diagnosis of spinal cord lesions to ensure prompt and appropriate management, which ultimately could enhance survival rates and the quality of life for these patients.

## Figures and Tables

**Table 1:** Age, sex, clinical presentation, management and outcome of primary cauda equina diffuse large B-cell non-Hodgkin lymphoma.

Case	Authors, Year (y)	Age (y)	Sex	Clinical Presentation	Management	Outcome
1	Mauney et al., 1983 [5]	68	F	Paraplegia, flaccid paralysis of lower extremities with muscular atrophy, deep tendon reflexes absent, and sensory loss up to the mid-thigh without return of bowel or bladder.	Myelogram, cerebrospinal fluid examination, bilateral laminectomy at T12-L3	Follow-up 3 months: no improvement of flaccid paralysis nor return of bowel or bladder
2	Toner et al., 1987 [6]	59	M	CES	Biopsy, myelogram, cerebrospinal fluid examination	Alive at 22 months
3	Klein et al., 1990 [7]	29	F	CES (AIDS)	Tumor resection	Fulminant lymphomatous meningitis and died
4	Giobbia et al., 1999 [8]	30	F	Cauda equina irritation	Cytology of CSF, RT treatment	Alive at 12 months
5	Zagami and Granot, 2003 [9]	71	F	Cauda equina irritation	8th thoracic laminectomy, CSF, intrathecal chemotherapy	Dead at 8 months
6	Tajima et al., 2007 [10]	67	F	Polyradiculoneuropathy	Biopsy, CSF intrathecal chemotherapy; RT	Alive at 36 months
7	Khong et al., 2008 [11]	16	M	CES	Biopsy, intravenous chemotherapy; RT	Alive at 12 months
8	Beitzke et al., 2010 [12]	69	M	Pronounced flaccid lower extremity paraparesis and severe low-back pain	Biopsy, positron emission tomography, cerebrospinal fluid examination, contrast-enhanced MRI	Died of septic shock soon after diagnosis
9	Teo et al., 2012 [13]	58	M	CES	Biopsy, intrathecal chemotherapy; RT	Alive at 24 months
10	Cugati et al., 2012 [14]	11	M	CES	Tumor excision, intrathecal chemotherapy; RT	Alive at 12 months
11	Iwasaki et al., 2012 [15]	69	M	CES	Biopsy, PET/CT, intravenous chemotherapy; RT	Dead at 18 months
12	Nishida et al., 2012 [16]	47	M	CES	CSF, PET/TC, intravenous and intrathecal chemotherapy, RT	Alive at 18 months
13	Nakashima et al., 2014 [17]	59	M	CES	Biopsy, chemotherapy, RT	Alive at 12 months
14	Broen et al., 2014 [18]	71	F	S1 radiculopathy	Biopsy, PET/TC intravenous and intrathecal chemotherapy	Alive at 10 months
15	Broen et al., 2014 [18]	75	F	L5 radiculopathy	Biopsy, steroid only	Dead after 11 months
16	Shin et al., 2016 [19]	79	F	CES	Biopsy	Alive at final follow-up
17	Belcastro et al., 2016 [20]	47	M	CES	Biopsy, intrathecal chemotherapy	Dead 2 months,
18	Suzuki et al., 2018 [21]	65	M	Progressive motor palsy in the legs and gait disturbance over the last 5 months; deep tendon reflexes of the bilateral lower extremities were diminished. Sensory disturbance was also found in the bilateral lower extremities.	Biopsy after laminectomy, positron emission tomography, cerebrospinal fluid examination, contrast-enhanced MRI, cytarabine and methotrexate (high dose)	Complete remission after 6 years

**Table 2:** Neuroradiological finding, histological classification, and side of primary cauda equina diffuse large B-cell non-Hodgkin lymphoma.

Case	Authors, y	Neuroradiological Finding	Histological Classification	Side
1	Mauney et al., 1983 [5]	Multiple intradural masses with a complete block of the subarachnoid space	B-cell malignant large-cell noncleaved (Lukes and Collins classification)	T12-L3
2	Toner et al., 1987 [6]	Intradural masses with a complete	B-cell large-cell	Lumbar nerve root
3	Klein et al., 1990 [7]	Swelling of cauda equina, T1WI focally high	-	L1-L2
4	Giobbia et al., 1999 [8]	Enhancement of the lesions on post contrast MRI, swelling of cauda equina	Diffuse large B-cell lymphoma (DLBCL)	L5-S1
5	Zagami and Granot, 2003 [9]	Enhancement of the lesions on post contrast MRI, swelling of cauda equina	Diffuse large B-cell lymphoma (DLBCL)	Below cornus
6	Tajima et al., 2007 [10]	Low T2WI, enhancement of the lesions on post contrast MRI, swelling of cauda equina	Diffuse large B-cell lymphoma (DLBCL)	T12-L3
7	Khong et al., 2008 [11]	Low T2WI, low T1WI, enhancement of the lesions on post contrast MRI, swelling of cauda equina	Diffuse large B-cell lymphoma (DLBCL)	T12-L3
8	Beitzke et al., 2010 [12]	Diffuse thickening of the cauda equina nerve roots or intradural nodular masses with enhancement of the lesions on post contrast MRI	Diffuse large B-cell lymphoma (DLBCL), massive diffuse interstitial infiltrate of atypical lymphocytic cells (expressed CD20, CD79, and S100) Ki-67 80–90%	Cauda equina nerve roots and the left S1 nerve
9	Teo et al., 2012 [13]	Swelling of cauda equina, slightly high T2WI	Diffuse large B-cell lymphoma (DLBCL)	T12-L3
10	Cugati et al., 2012 [14]	Swelling of cauda equina, isointense T1WI and T2WI	large B-cell lymphoma	T11-L4
11	Iwasaki et al., 2012 [15]	Swelling of cauda equina, slightly high T2WI, enhancement of the lesions on post contrast MRI	Diffuse large B-cell lymphoma (DLBCL)	T12-L1
12	Nishida et al., 2012 [16]	Low T1WI, slightly high T2WI, swelling of cauda equina, enhancement of the lesions on post contrast MRI, increased FDG accumulation	Diffuse large B-cell lymphoma (DLBCL)	T12-L2
13	Nakashima et al., 2014 [17]	Low T1WI, low T2WI, swelling of cauda equina, enhancement of the lesions on post contrast MRI, increased FDG accumulation	Diffuse large B-cell lymphoma (DLBCL)	T12-S1
14	Broen et al., 2014 [18]	Low T2WI, swelling of cauda equina, enhancement of the lesions on post contrast MRI, increased FDG accumulation	Diffuse large B-cell lymphoma (DLBCL)	L2-L5
15	Broen et al., 2014 [18]	Low T2WI, swelling of cauda equina, enhancement of the lesions on post contrast MRI	Diffuse large B-cell lymphoma (DLBCL)	T12-L5
16	Shin et al., 2016 [19]	Low T2WI, swelling of cauda equina, enhancement of the lesions on post contrast MRI	Diffuse large B-cell lymphoma (DLBCL)	L3-L5
17	Belcastro et al., 2016 [20]	Swelling of cauda equina, enhancement of the lesions on post contrast MRI, increased FDG accumulation	Diffuse large B-cell lymphoma (DLBCL)	L2-L4
18	Suzuki et al., 2018 [21]	Enlargement of the cauda equina occupying the dural sac from the L1-S1 level with isointensity to the spinal cord signal on both T1- and T2-weighted imaging; diffuse accumulation of 2-fluoro-2-deoxy-glucose was observed in the cauda equina	Diffuse large B-cell lymphoma (DLBCL), non germinal center (expressed CD20, BCL2, BCL6, and MUM-1)	L4-L5

## Data Availability

The data that support the findings of this study are available from the corresponding author, upon reasonable request.
